# Innovative methods to analyse the impact of gender norms on adolescent health using global health survey data

**DOI:** 10.1016/j.socscimed.2021.114652

**Published:** 2022-01

**Authors:** Beniamino Cislaghi, Ann M. Weber, Holly B. Shakya, Safa Abdalla, Amiya Bhatia, Benjamin W. Domingue, Iván Mejía-Guevara, Lindsay Stark, Ilana Seff, Linda M. Richter, Ana Maria Baptista Menezes, Cesar G. Victora, Gary L. Darmstadt

**Affiliations:** a London School of Hygiene and Tropical Medicine, Department of Global Health and Development, London, UK; b School of Public Health, University of Nevada, Reno, NV, USA; c Department of Medicine, Center on Gender Equity and Health, University of California, San Diego, La Jolla, CA, USA; d Global Center for Gender Equality, Department of Pediatrics, Stanford University School of Medicine, Stanford, CA, USA; e Graduate School of Education, Stanford University, Stanford, CA, USA; f Center for Population Health Sciences, Stanford University School of Medicine, Stanford, CA, USA; g Stanford Aging and Ethnogeriatrics (SAGE) Research Center, Stanford University School of Medicine, Stanford, CA, USA; h Brown School of Social Work, Washington University in St. Louis, St. Louis, MO, USA; i Centre of Excellence in Human Development, University of Witwatersrand, Durban, South Africa; j International Center for Equity in Health, Postgraduate Program in Epidemiology, Federal University of Pelotas, Pelotas, Rio Grande de Sul, Brazil

**Keywords:** Gender norms, Global health, Survey data, Gender equality, Attitudes, Behaviours, Reference group, Sanctions

## Abstract

**Background:**

Understanding how gender norms affect health is an important entry point into designing programs and policies to change norms and improve gender equality and health. However, it is rare for global health datasets to include questions on gender norms, especially questions that go beyond measuring gender-related attitudes, thus limiting gender analysis.

**Methods:**

We developed five case studies using health survey data from six countries to demonstrate approaches to defining and operationalising proxy measures and analytic approaches to investigating how gender norms can affect health. Key findings, strengths and limitations of our norms proxies and methodological choices are summarised.

**Findings:**

Case studies revealed links between gender norms and multiple adolescent health outcomes. Proxys for norms were derived from data on attitudes, beliefs, and behaviours, as well as differences between attitudes and behaviours. Data were cross-sectional, longitudinal, census- and social network-based. Analytic methods were diverse. We found that gender norms affect: 1) Intimate partner violence in Nigeria; 2) Unhealthy weight control behaviours in Brazil and South Africa; 3) HIV status in Zambia; 4) Health and social mobility in the US; and 5) Childbirth in Honduras.

**Interpretation:**

Researchers can use existing global health survey data to examine pathways through which gender norms affect health by generating proxies for gender norms. While direct measures of gender norms can greatly improve the understanding of how gender affects health, proxy measures for norms can be designed for the specific health-related outcome and normative context, for instance by either aggregating behaviours or attitudes or quantifying the difference (dissonance) between them. These norm proxies enable evaluations of the influence of gender norms on health and insights into possible reference groups and sanctions for non-compliers, thus informing programmes and policies to shape norms and improve health.

## Highlights

• This article presents effective methods to study gender norms in existing global health survey data.
• We devised conceptual pathways linking gender norms to gender-based health disparities.
• We identified gender norms proxies and reference groups enforcing the norm.
• We tested hypotheses linking gender norms to health.
• These methods can aid policy and programme design to advance gender equality and health.

## 1 Introduction

Unprecedented attention is being directed to understanding how gender influences health-related outcomes (Heise et al., 2019). While sex refers to the biological features that contribute to making a person male, female or intersex, gender reflects the social system of beliefs, expectations, and roles that are assigned to people because they are men, women or gender minorities (Heise et al., 2019). Gender inequities begin before birth and persist across the life course, influencing manifold aspects of people's lives, including one's chances of being born (Hesketh, 2011), developing specific health conditions (Cislaghi et al., 2020), and being taken to the hospital (Weber et al., 2019).

Understanding how gender norms affect people's health can help inform policies and programmes to reduce gender-based health inequities (Weber et al., 2019; Cislaghi and Heise, 2020). Norms can be descriptive (one person's beliefs of what others in the group do) or injunctive (one person's beliefs of the extent to which others in one's group approve of something) (Cialdini et al., 1990). Despite the need to respond to harmful effects of gender norms on health, global health surveys rarely include gender norms data (Weber et al., 2021). Moreover, gender norms generally are described qualitatively, and rarely are there attempts to quantify the effects of norms on health outcomes (Heise and Kotsadam, 2015). Recently, we developed proxies and methods to investigate existing global datasets for insights into how gender norms can affect health, and furthermore, to quantify those effects. These methods and proxies were used to generate the findings presented elsewhere (Weber et al., 2019). In this paper, instead, we detail the methodological approaches we used in five case studies. We examine the roadblocks encountered and how we overcame them to offer global health researchers a framework for empirical analysis of gender norms and health.

## 2 Methods and results

### 2.1 Case studies

**Fig. 1:**
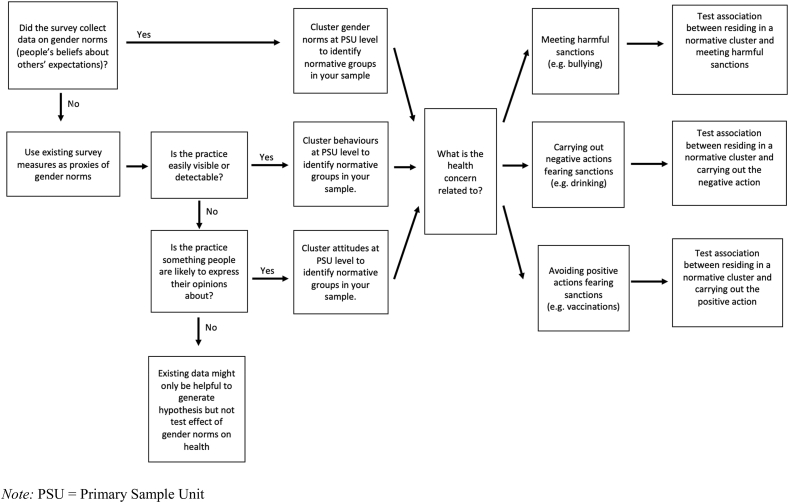
Decision-making flowchart for constructing gender norms proxies.

Table 1, Table 2, Table 3, Table 4, Table 5 provide information about the context and data, health outcomes, gender norms and health hypothesis, gender norms proxy measures, analytic approach, findings and reflections on the strengths and limitations of the methodological approach used for each of the case studies to illustrate key principles and approaches to gender norms analysis (Weber et al., 2019; Domingue et al., 2019; Shakya et al., 2019, 2020). Here we focus on methodological strategies and approaches which we synthesise into a decision-making flowchart for researchers to facilitate the construction of gender norms proxies for use in future research (Fig. 1). Case studies were selected through an iterative process. We relied on (1) data availability (there being sufficient sample sizes and appropriate measures), (2) existing analytical methods that we could use to analyse data from the dataset, and (3) the appropriateness of the case study to link to a conceptual framework for pathways through which gender norms impact health. We also sought to identify a selection of cases studies which illustrate a diversity of contexts and health outcomes for which the relevance of gender norms for health could be quantified.

**Table 1:** Case Study: Gender norms for labour force participation and violence against adolescent girls in Nigeria.

Country	Nigeria
**Dataset**	2014 Violence Against Children Survey
**Sample**	Cross-sectional survey of 13–24-year-old females (n = 1633)
**Health outcomes of interest**	Intimate partner violence
**Hypothesis**	Husbands of women who work for pay in places where that is counter-normative will be ridiculed and will respond by being violent with their wives.
**Gender norms proxy**	Cluster-level female participation in paid labour
**Analysis methods**	Step 1: Intraclass correlation coefficient (ICC) to examine clustering of attitudes towards female labour employment (revealed attitudes as inadequate proxies). Step 2: Empirical identification of cut-off point as normative threshold for women's participation of labour employment (communities with <30% of women participating in paid labour were labelled as counter-normative – i.e. with a norm against women's participation). Step 3: Logistic regressions to compare experience of violence of women living in communities below and above normative threshold.
**Results (summary)**	We found no differences in overall rates of past-year IPV between the community types, with approximately 7–8% of girls in each community reporting physical or sexual IPV in the last 12 months (Weber et al., 2019). However, results showed that, in communities with restrictive norms against FLP, girls who worked outside the home were significantly more likely to experience IPV as compared to those who did not work outside the home; this same relationship was not observed in communities where FLP was normative. Thus, women who engaged in paid labour in contexts where female participation in paid labour was counter-normative were more likely to experience IPV, possibly as a sanction against their counter-normative behaviour (that is, behaviour contradicting the social norm).
**Strengths and limitations**	Strengths: The ICC can be a powerful tool to determine whether the attitude or behaviour of interest is under normative influence. ICC can also be used to identify a reference group which is enforcing gender norms. Limitations: When the ICC for a given attitude or behaviour is low for certain groups, constructing a group-level norm proxy from this attitude or behaviour may not be appropriate, as was the case with attitudes around IPV as discussed above. While the outcome in this case study refers to the past year (past-year IPV victimisation), the predictor references the past week (FLP in the past week). Ensuring temporality in the data between the predictor and outcome of interest would help others conducting similar analyses in the future. Further research is needed to identify an ICC threshold that more confidently implies normative clustering. However, by identifying a threshold of community-level FLP, we were able to test for an impact of transgressing the norm.

**Table 2:** Case Study: Body gender norms and adolescent mental health in Brazil and South Africa.

Country	Brazil; South Africa
**Dataset**	Pelotas Birth Cohort Study 1993, Brazil, longitudinal Birth-to-Twenty-Plus cohort study, South Africa, longitudinal
**Sample**	Longitudinal cohort data on infants born in Pelotas City, Brazil in 1993, followed-up at ages 11, 15, and 18 years. Longitudinal cohort data on infants born in 1990 in Johannesburg, South Africa in 1990, followed-up at ages 13, 17, and 22 years.
**Health outcomes of interest**	Mental health in Brazil, measured by the Strengths and Difficulties Questionnaire (SDQ) score. Eating disorders risk in South Africa, measured by the Eating Attitudes Test (EAT) score.
**Hypothesis**	Brazil: Adolescents' perception of their parents thinking they are thin or fat at age 11 affected their mental health at age 18, and was moderated by body dissatisfaction. South Africa: Adolescents' perception of their peers' approval of their bodies influenced their eating disorders risk at age 22, mediated through body dissatisfaction.
**Gender norms proxy**	Brazil: Normal BMI adolescents' perception of their parents' opinion about their weight. South Africa: Adolescents' perception of their peers' approval of their appearance.
**Analysis methods**	Brazil: Tobit regression of the SDQ scores at age 18 on body dissatisfaction at age 15, examining a potential moderating role for adolescents' perception of their parents' opinion about their weight at age 11. South Africa: Mediation analysis with linear regression of change in eating disorder scores over time as the outcome on change in peers' approval scores over time as the predictor, considering change in body satisfaction scores over time as the mediator.
**Results (Summary)**	Brazil: Girls who felt fatter than ideal at age 15 had higher risk of poor mental health at age 18, but only if they thought their parents judged them to be fat at age 11 (Weber et al., 2019). We found no similar association among boys (independently of the family norm) or among girls who at age 11 believed their parents judged them of normal weight or thin. Thus, BMI-normal girls whose parents unfairly judged them to be fatter than ideal experienced higher risk of poor adolescent mental health. South Africa: Stronger perception of approval by peers during adolescence was associated with greater body satisfaction among girls (Weber et al., 2019). In turn, body satisfaction was associated with a decrease in eating disorders risk scores. Thus, girls whose peers judged them as fatter as ideal were more likely to experience decreased eating disorder. Wealth played a moderating role in the association between body satisfaction and eating disorders risk among boys, with a similar indirect association between peers' approval and eating disorder risk scores for dieting more evident as wealth decreased.
**Strengths and Limitations**	Strengths: The normative influence variables we used allowed us to capture adolescents' beliefs of what others (parents, peers) thought of them, which, in this case, operated as an excellent gender norms measure. Limitations: The inability to test multiple influences in each of the studies, for example both parents and peers, limited the capacity to assess their relative importance or their interaction. Further research could investigate how different groups can exert different influences on adolescents' body weights, paving the way to understanding which groups in which settings should be the primary focus of interventions and policies.

**Table 3:** Gender norms toward pre-marital sex and risk of adolescent HIV in Zambia.

Country	Zambia
**Dataset**	2007 Demographic and Health Survey
**Sample**	Cross-sectional survey of 15–24-year-old males and females (n = 2954) and 25-49-year-old males and females (n = 7608).
**Health outcomes of interest**	Rate of HIV infection
**Hypothesis**	Unmarried sexually active adolescents in places where premarital sex is counter-normative (i.e. places where people hold attitude against it, but still do it) will not seek medical advice or help.
**Gender norms proxy**	Cluster-level attitudes towards adolescents' premarital sex
**Analysis methods**	Step 1: Contrast of cluster-level reported attitudes and cluster-level data-derived behaviours. Step 2: Sex-stratified Poisson regression to test association between adult discordance between attitudes and behaviours and adolescents' HIV status. Step 3: Sensitivity analysis to assess the separate effect of attitudes and behaviours.
**Results (summary)**	More than 80% of men or women disapproved of sex before marriage, but women were calculated as less likely to engage in premarital sex (51%) than men (89%) (Weber et al., 2019). In addition, both women and men's discordance of attitudes and behaviours were positively associated with adolescent women HIV prevalence at the regional level (Pearson correlation~60%). In individual fully adjusted models, a 10% increase in the discordance of adult women or men was associated with a 27% (RR = 1.27, 1.11–1.45; p = 0.001) or 28% (RR = 1.28, 1.05–1.56, p = 0.015), respectively, increase in individual relative risk of HIV infection for adolescent women. This relationship was not significant for risk of HIV infection in adolescent boys. Thus, the wider the taboo gap between stated attitudes and actual behaviour of premarital sex, the more likely adolescent girls are to be HIV positive.
**Strengths and limitations**	Strengths: We were able to use a derived measure of premarital sexual behaviour, thus potentially minimizing reporting bias, and also avoided the use of adolescents' data with potentially heightened bias in self-reported attitudes toward premarital sex. The use of discordance of attitudes and behaviours to assess non-compliance with norms could potentially be applied to a number of other issues where discordance between self-reported attitudes and actual behaviour might create a taboo gap that, in turn, would reduce seeking help and disclosing sanctioned behaviours. This method can also have useful applications in evaluating and designing purposeful interventions. As an example, if an intervention were to be planned in Zambia on reducing HIV prevention, it would be important to address the taboo gap that inhibits seeking contraceptive care, especially in communities where the gap is stronger. Attitudinal data was available with a good balance by sex and age that allowed the calculation of differences of adults' attitudes and behavioural sex, separately for adolescents and adults. Limitations: We currently lack reliable community-level gender norms data across countries and time that would allow a better assessment of the community-level effects and trends, ultimately improving research and action. We only identified six countries containing information on attitudes towards premarital sex and adolescents' HIV status: Cambodia, Dominican Republic, Niger, Sao Tome and Principe, Swaziland, and Zambia. Zambia was the only country with reliable attitude measures across regions, however, with the large majority of both men and women uniformly denouncing premarital sex.

**Table 4:** Gender expression norms and adolescent health and social mobility in the US.

Country	United States
**Dataset**	National Longitudinal Study of Adolescent to Adult Health
**Sample**	Waves of cross-sectional data collected from 11 to 18-year olds (wave 1, 1994–1995, n = n = 20,74) who are followed into young adulthood (wave 4, ages 24–32 years, (N = 15,701)
**Health outcomes of interest**	Age of first sexual intercourse; alcohol use; attempted suicide; delinquent behaviour; depressive symptoms; drug use; suicidal ideation; self-reported health; weight-gain behaviours; weight-loss behaviours
**Hypothesis**	Adolescents who behave “outside of the norm” experience worst health overall and lower social mobility
**Gender norms proxy**	Mean gendered behaviour for respondents' same-sex, same-grade, same-school peers
**Analysis methods**	Step 1: Measured probability participants' actions matched the statistical norms of actions carried out by other same-sex, same-school, same-year participants. Step 2: Construction of school-level gender norm by calculating mean gendered behaviour for same-sex, same-school, same-grade peers Step 3: Associations between health-related behaviours and alignment between participants' gendered actions and statistical norms of gendered actions
**Results (summary)**	Exposure to more masculine grade-level peers was associated with reduced levels of educational (Standardized Association [SA] = -1.04, [-1.553, −0.519], p < 0.0001) and occupational attainment for males (SA = −0.848, [-1.381,-0.315], p = 0.0018] (Heise and Kotsadam, 2015). In contrast, the gender normativity of a female's same-sex grade-level peers was unassociated with later educational (SA = 0.517, [-0.002,1.036], p = 0.05) and occupational attainments (SA = 0.386, [-0.228,0.999], p = 0.22). Boys and girls who reported more typically masculine behaviours than their same-sex peers were significantly more likely to report risky behaviours, for example engaging in delinquent behaviour (β = 0·158, 95% CI 0·128–0·187, p < 0·0001 for girls and β = 0·399, 95% CI 0·345–0·454, p < 0·0001 for boys) (Weber et al., 2019). Conversely, boys and girls who reported more typically feminine behaviours were more likely to report weight-loss behaviours (β = 0·228, 95% CI 0·180–0·276, p < 0·0001 for girls and β = 0·143, 95% CI 0·107–0·179, p < 0·0001 for boys). Girls were more likely to report increased depressive symptoms and ideation and attempts at suicide with increasing difference in either direction (more typically masculine or feminine) from the median gender normativity score of their peers. In men, higher masculine gender expression in adolescence was positively associated with several health behaviours and outcomes in adulthood, including having smoked in the previous 30 days, having ever used marijuana or recreational drugs, fast food and soda consumption in the previous week, better self-rated health, a lower likelihood to attempt to lose weight, and more physical activity (Domingue et al., 2019). In women, higher feminine gender expression in adolescence was positively predictive of a number of health outcomes and behaviours in adulthood, including high cholesterol, physical limitations, ever having used recreational drugs, ever having misused prescription drugs, experience of sexual violence, obesity, and depression.
**Strengths and limitations**	Strengths: The use of a gender expression measure to study norms could be extended to other settings. The key data requirement is a battery of attitudes, beliefs and behaviours that can be used to predict self-reported sex. The measure itself can be used, as we have demonstrated, in various ways. As a stand-alone measure, it represents level of adherence to overall trends of “masculine” and “feminine” behaviour; aggregated at the school-specific grade level, it reflects local normative context, and the difference between the individual measure and local context adds the dimension of how much any individual deviates from the local norms. The basic framework of this measure could also be used to study other types of norms. For example, in the Add Health data, one could imagine studying norms within special groups (by SES, education, race, geographical location) using a similar design. Limitations: Our measure is a proxy - it does not measure any individual's perceptions of what is normative, or the possible sanctions if they do not adhere. However, because self-reported norms can, like any self-reported measure, be weakened by response bias. Alternative methods of measuring norms, like these, are an important contribution to this body of research.

**Table 5:** Social network and community norms on adolescent childbirth in Honduras.

Country	Honduras
**Dataset**	Honduras randomised controlled trial dataset
**Sample**	Census and social network data on adolescent girls 15–20 years old
**Health outcomes of interest**	Adolescent childbirth
**Hypothesis**	Social network factors would be relevant determinants of having had an adolescent childbirth
**Gender norms proxy**	Norms and behaviours related to adolescent childbirth
**Analysis methods**	Social network analysis
**Results (summary)**	The chance that a girl had an adolescent childbirth was much higher if she was socially connected to someone who had had an adolescent childbirth, particularly if she had a strong relationship with that person, or they were close in age (Shakya et al., 2019). The village-level aggregate adolescent childbirth measure was also strongly associated with a girl's likelihood of having been an adolescent mother. However, the addition of the village-level aggregate variable to the model did not attenuate the association between individual social contacts' adolescent pregnancy and the girl's childbirth, suggesting that both interpersonal descriptive norms and community-level descriptive norms were important factors associated with adolescent childbirth. A girl was also more likely to have had an adolescent childbirth if her important social contacts believed that the village supported adolescent childbirth. This association, however, was attenuated when the village aggregate normative measure was included in the model, suggesting that the larger context of the village is the main source of injunctive normative pressure.
**Strengths and limitations**	Strengths: Social network analysis is a powerful tool for understanding the direct connections between individuals, the people in their networks, and the expectations and associations regarding behaviours in those networks. In this analysis we had full census data from 176 villages, with social network data on most community members, allowing us to see the connections between individuals and their shared norms and behaviours within each of these communities. Social network data of this sort allows us to construct the social architecture of norms and behaviours: with first-hand reports from all participants and the detailed social connections between them. In the absence of social network data, interpersonal influences cannot be measured and reliance must be placed on aggregate-level or clustered beliefs and behaviours, which sacrifices precision. Limitations: Our village-level aggregate measures, however, were far more comprehensive than those usually used in similar analyses, which tend to aggregate measures from a sampled sub-section of the population. While our injunctive norms question directly measured individuals' perceived norms, our descriptive norms measures - adolescent births at the interpersonal and aggregate community levels - were proxies. A major limitation of this analysis was the cross-sectional nature. Ideally, we would want to follow girls over time, using norms within their networks and communities to predict pregnancies. Instead, we had to take an event that had occurred in the past and look at associations with current measures of norms. While village-level aggregate outcomes are unlikely to change as a result of any one girl's pregnancy outcomes, interpersonal norms and attitudes may.

#### 2.1.1 Case study 1. Gender norms for labour force participation and violence against adolescent girls in Nigeria

Reported attitudes or behaviours, particularly when clustered and variable across primary sample units (PSU), can signal the presence of norms and provide an opportunity to explore quantitatively their effects on health.

##### 2.1.1.1 Motivation

Using cross-sectional data from Nigeria’s 2014 Violence Against Children Survey (NVACS), we explored the relationship between female labour participation (FLP) and intimate partner violence (IPV) victimisation for adolescent girls, assessing how this relationship might be modified by FLP norms at the PSU level (in this case, informal settlements or “communities” of variable population size) (Table 1). NVACS utilised a multi-stage cluster sample design and is nationally representative of females 13–24 years old (n = 1633) (Stark et al., 2020). Survey data verified that male adolescents (13–17 years) [68%, 95% confidence interval (CI) 64–72%] and young adults (18–24 years) (83%, 95% CI 80–86%) were significantly more likely than female adolescents (46%, 95% CI 40–52) or young adults (56% 95% CI 51–61) to report engaging in work (UNICEF, 2016). Moreover, among those who worked, adolescent (59%) and young adult (40%) girls most commonly worked in a family dwelling whereas adolescent (51%) and young adult (40%) boys worked most commonly in an outside farm or garden. Gender norms that promote male dominance have been shown to sustain IPV (Heise, 1998) as well as the expectation that men should work outside the household while women should not (Anyanwu and Augustine, 2013; Gage and Thomas, 2017). We hypothesised, consistent with existing literature, that women who transgress this norm might face IPV (Hynes et al., 2016; Manji et al., 2020). Our primary outcomes of interest included physical IPV and sexual IPV. The predictor of interest, FLP, was defined as participating in formal or informal work outside the home in the last week. All measures were self-reported.

##### 2.1.1.2 Methodological roadblocks and solutions

We sought to take advantage of the clustered nature of the NVACS data, initially using data on attitudes toward acceptance of IPV, aggregated across communities (primary sample units in the survey, most often villages). Doing so, however, generated no meaningful results, suggesting that uniform normative attitudes toward IPV may be pervasive (Chae et al., 2020). We then applied the Intraclass Correlation Coefficient (ICC – commonly used to quantify the resemblances between individuals with a fixed degree of relatedness) to examine whether these attitudes were clustered within communities. In general, higher ICCs demonstrate greater clustering within and variability across communities, in turn implying that individuals’ attitudes are normatively influenced by the attitudes of others in the community (Legros and Cislaghi, 2019). ICCs for all explored attitudes across communities were found to be low, pointing to attitudinal heterogeneity and suggesting that attitudes around IPV were not under normative influence within communities. Next, we examined and found clustering of FLP, a behaviour. Rather than using the proportion of women in each community who engaged in FLP as an explanatory factor of IPV, we let a natural cut-point emerge from the data for a normative threshold of community-level FLP (Centola et al., 2018). We plotted the distribution of FLP across communities and found that, in two-thirds of communities, fewer than approximately one-third of young women and adolescent girls in those communities participated in the labour force, i.e., communities where FLP was <28% corresponded to the 66th percentile. These communities were thus classified as those where FLP was not a social norms. Interestingly, this cut-point aligns with recent social norms experiments that found 25% to be the critical threshold for norm maintenance or change within a community (Centola et al., 2018).

Using adjusted Wald tests, past-year victimisation of physical and sexual IPV were first compared across the two community types: restrictive (<28% of women in the labour force) and non-restrictive (≥28% of women worked outside the home) with regard to FLP. To examine how the relationship between working outside the home and risk of violence might be modified by community norms around FLP, logistic regressions were used to estimate violence outcomes of interest stratified by the two community types. Regressions controlled for a girl's FLP outside the home, marital status, age, and ever having attended school. All observations were weighted to be representative of the population of 13–24 year-old females in Nigeria and standard errors were adjusted for the multi-stage sampling design. This case study demonstrated a novel use of the ICC for a behaviour as a promising avenue for proxying norms.

The flow chart in Fig. 1 could have helped us make the following decisions: Because the survey did not collect data about expectations of others, we had to develop a gender norms proxy. The practice of working outside of the household was visible, thus we could cluster attitudes at the provincial level. Because the health concern was related to avoiding a positive action (working outside of the household) for fear of sanctions (violence), we tested associations between residing in a normative cluster and carrying out the positive practice of interest.

#### 2.1.2 Case study 2. Body gender norms and adolescent mental health in Brazil and South Africa

Longitudinal data provides a powerful opportunity to construct and quantitatively test pathways linking gender norms to health outcomes – a critical component of gender norms analysis.

##### 2.1.2.1 Motivation

Using longitudinal birth cohort data from Brazil (Gonçalves et al., 2014), we investigated the pathway between early exposure to gender norms related to body image and mental health later in adolescence (Table 2). Additionally, we used longitudinal data from a majority Black birth cohort in an urban area in South Africa to investigate how exposure to gender norms in adolescence affected the risk of eating disorders in early adulthood (Richter et al., 2007). Body image-related norms are highly gendered, with an emphasis on thinness for girls and muscularity for boys (Karazsia et al., 2017). Recent evidence suggests that with urbanization and exposure to Western media and rising obesity prevalence, norms on thinness are increasingly emerging in African countries, including South Africa, and traditional and emerging norms often co-exist (Fadipe et al., 2017; Cohen et al., 2020). Research on normative influence on body dissatisfaction and its relation to weight control behaviours and mental health is heavily skewed towards western cultures or illustrates only short-term impacts where these norms were found to be enforced and/or propagated by parents, peers and the media (Ricciardelli et al., 2003). We hypothesised that longitudinal data from alternative global settings would enable us to gain new insights into ways in which social norms on appearance influence body dissatisfaction and subsequent adolescent weight control behaviours and mental health.

##### 2.1.2.2 Methodological roadblocks and solutions

Cohort data from Brazil included variables related to what adolescents believed to be their parents' judgments of their own weight (we called this variable “family gender norms”). The dataset also included variables on which school they attended. We attempted to identify school-level clustering of body weight or body-related attitudes to capture norms among peers but found none. We settled on using gender norms in the family, although we were challenged to distinguish parents' unfair judgements towards healthy children from situations where parents were worried about their child's unhealthy body weight. To overcome this hurdle, we isolated a subset of adolescents with normal Body Mass Index (BMI) at age 11. We tested whether perceived parents' opinion of their adolescent's weight at age 11 modified the impact of body dissatisfaction on mental health at age 18 as measured by the Strengths and Difficulties Questionnaire (SDQ) (Goodman, 1997). To increase the validity of our analysis, we adjusted for body dissatisfaction and mental health status at age 11.

In the South African dataset, we identified two questions that had potential as proxies for gender norms: 1) which celebrities did adolescents want to look like, and 2) whether adolescents believed their friends approved of their bodies. Using the former variable was attractive, as it would have allowed an investigation of how media messages about body types could affect adolescents' weight control practices. Unfortunately, there were too many missing values. The latter measure, which we refer to as “peer gender norms,” provided the opportunity to explore the normative influence of peers on adolescent weight control behaviour. We conducted linear regressions to investigate the relationship between changes in perception of peers' approval of adolescents' bodies and the eating disorder risk score (measured by the Eating Attitudes Test) (Garner et al., 1982) of adolescents between ages 13–17, adjusting for change in BMI between ages 13–17, and looking at a mediating role of change in body satisfaction. Using these two datasets (Brazil and South Africa), we were able to explore multiple proxies for examining pathways for the influence of body norms held by multiple reference groups (parents, peers, media) on adolescents’ weight control and eating behaviours.

The flow chart in Fig. 1 could have helped us make the following decisions: Because the survey did collect data about expectations of others, we clustered norms data at the PSU level. Because the health concern was related to carrying out a negative action (eating habit) fearing sanctions (not being liked), we tested associations between residing in a normative cluster and carrying out the negative practice of interest.

#### 2.1.3 Case study 3. Gender norms toward premarital sex and risk of adolescent HIV in Zambia

While data on attitudes or behaviours can be useful in gender norm analysis, the difference or “discordance” between them can provide novel insights into normative processes in communities and their effects on health.

##### 2.1.3.1 Motivation

Using cross-sectional data from the 2007 Zambia Demographic and Health Survey (ZDHS), we examined the effect of gender norms towards premarital sex on sexually active adolescents’ HIV status (Table 3). Gender norms are considered critical for understanding premarital sex attitudes and behaviours (Zuo et al., 2012). While we know that restrictive gender norms can lead to adverse sexual and reproductive health outcomes and risk behaviours (Jewkes et al., 2010), little is known about how proscriptive gender norms that stigmatise premarital sex can increase the risk of HIV infection among sexually active unmarried adolescents.

Our analysis examined whether gender norms proscribing premarital sex could increase HIV risk by discouraging sexually active adolescents in Zambia from reaching out for help on sex-related issues. We were interested in whether this, as we called it, “taboo gap” (the gap between professed attitudes and recorded behaviours) could increase adolescents’ risk of HIV infection. We expected a positive association as we hypothesised that the larger the gap between stated attitude and actual behaviour – which generates greater perceived risk of social sanction against the behaviour and lowers the likelihood that adolescents would seek out HIV-protecting methods (Weber et al., 2019) – the greater the risk for acquiring HIV through unprotected sex.

##### 2.1.3.2 Methodological roadblocks and solutions

While there were 16 countries which reported information on attitudes towards premarital sex, only six countries collected information on adolescents' HIV status. Among these, Zambia was the only country with sufficient within-country variation in self-reported attitudes toward premarital sex to study their impact on HIV infection risk. In the absence of an explicit measure of norms towards premarital sex and to develop a proxy for the taboo gap, we first constructed a measure of group-level self-reported attitudes related to premarital sex. Because DHS data lacks representativeness of primary sampling units (∼25 households per community), we used geographic provinces (subdivided into urban or rural) as clusters. Aggregate attitudes (measured as responses to the item “should young women/men wait for sex until marriage?“) were approximated by separately averaging individual attitudes of adult female and male residents (aged 25–49 years) in 18 urban and rural strata (i.e., provinces or clusters) available in the ZDHS. Then we sought to develop cluster-level estimates of the extent to which the stated attitude was not respected. Since ZDHS does not include direct questions about premarital sex, we used adults' self-reported age at first sexual intercourse and age at first marriage to derive an estimate of adults who had sex before marriage. We then calculated weighted aggregate differences between reported attitudes and derived behaviours at the cluster (provincial) level. Attitudinal data was available with a good balance by sex and age that allowed the calculation of differences of adults' attitudes toward and behaviours of engaging in premarital sex. We used the difference between adults' attitudes and behaviours as representative of the reference group in communities which potentially enforces sanctions for non-compliance on the part of adolescents, thus generating the taboo gap. Next, we tested the effect of adults' discordance between premarital sex attitudes and behaviours on the risk of HIV acquisition in adolescents using sex-stratified Poisson regression models, controlling for adolescent demographic characteristics and community-level factors and for the cluster survey design of ZDHS. To our knowledge, this type of analysis offered an innovative method as it used adults' discordance in premarital sex attitudes and behaviours as a proxy for the normative forces that shape adolescent sexual behaviours and risk for HIV. By using data on adult premarital sex attitudes and behaviours, we also avoided possible bias in adolescents’ self-reports on a sensitive and private issue. Finally, it is worth noting that, because of the social norm against premarital sex, some respondents could have avoided reporting a date before marriage for their first sexual intercourse. Because, nevertheless, we still found relevant discordance, it could be hypothesised that the real discordance is even larger.

The flow chart in Fig. 1 could have helped us make the following decisions: Because the survey did not collect data about expectations of others, we had to develop a gender norms proxy. Because the practice (premarital sex) was largely undetectable, we clustered behaviours at the provincial level and their mismatch with people's attitudes. Because the health concern was related to avoiding a positive action (using contraception and accessing sexual health services) fearing sanctions from parents, we tested associations between residing in a normative cluster and carrying out the negative practice of interest, looking at its effect on adolescent's HIV status.

#### 2.1.4 Case study 4. Gender expression norms and adolescent health and social mobility in the US

Identifying normative bounds – in this case based on a mix of multiple attitudes, beliefs and reported behaviours – and a reference group which can sanction behaviours outside the norm can yield powerful insights into effects of gender norms on health.

##### 2.1.4.1 Motivation

Using data from the National Longitudinal Study of Adolescent to Adult Health (Add Health) (Harris et al., 2019), we evaluated the effects of prevailing gender norms among adolescents in US schools (Table 4). The Add Health survey first selected a representative sample of over 100 schools across the US and then sampled students from within schools; this clustering of respondents into relatively proximate groups allowed us to make inferences about the gender norms amongst one's social peers. Add Health, like many large nationally representative surveys, does not include explicit measures of gender norms. However, a proxy measure was developed that used gender typical attitudes, beliefs and reported behaviours across the entire dataset to calculate the degree to which adolescents express themselves in a way that is typical of others in their school and grade, i.e., same-sex peers in their age cohort (Weber et al., 2019; Domingue et al., 2019; Shakya et al., 2019; Fleming et al., 2017). We hypothesised that deviation from norms for gender expression would be associated with adverse health outcomes and lower social mobility.

##### 2.1.4.2 Methodological approach, roadblocks and solutions

To construct our gender norms proxy, we identified a set of 26 self-reported questions that captured attitudes, beliefs and behaviours that were the most highly segregated by self-reported sex across the sample (see Supplementary Table 1) (Weber et al., 2019; Domingue et al., 2019; Shakya et al., 2019; Fleming et al., 2017). For each school, we used logistic regression with self-reported sex as the outcome, and the set of 26 variables as the predictors. To fit the model for each school, we used responses from students from outside that school to ensure predictions were not overly influenced by idiosyncrasies within each school being modeled. Using the model coefficients from the logistic regression and each individual's reports on the 26 measures, we estimated the predicted probability for each student within each school of falling within their self-reported sex. We described the resulting probabilities as a measure of gender expression. Large values (i.e., higher probabilities) represent respondents whose stated attitudes, beliefs and behaviours resulted in a strong model-based probability that they were the sex they reported to Add Health, i.e. more gender typical. Smaller values represent more gender-atypical adolescents who express themselves in more masculine or feminine ways than their same reported sex peers. This variable on its own provides insight into gender normativity: each respondent's value is a measure of how gender typical they are within the larger population of adolescents across the US.

The measure of gender expression, however, does not capture the degree of gender normativity within the individual students' more proximal context, where sanctions for behaviours outside the norm are more likely to be experienced. To capture that degree of normativity, we aggregated measures of gender expression for same-sex school peers of the respondent who were also in the same school grade and same class/year. Based on a precedent of aggregating behaviours as descriptive norms (e.g., see case reports 1, 3, 5), we deemed that gender expression was an appropriate measure to aggregate for use in measuring associations with downstream health, health behaviours and socioeconomic attainments (Weber et al., 2019; Domingue et al., 2019; Shakya et al., 2019; Fleming et al., 2017). We explored the use of the gender expression measure in several different ways. First, we used the individual measure of gender expression, controlling for grade-level norms. These analyses allowed us to explore whether a general tendency to behave in ways that are socially more or less masculine or feminine are associated with long-term health outcomes. By focusing on the within-school variation of gender norms across grades as a key point of leverage (Sotoudeh et al., 2019), we could ascertain how long-term social mobility was impacted. This measure allowed us to quantify the overall degree of adherence to what we operationalised as masculine and feminine in an adolescent's social context. Schools – and, consequently, the norms within them – vary for a large set of interrelated structural factors. If we focused strictly on associations between school norms and later behaviours, these structural factors could act as an unexplained confounder. However, within a school, the variation in gender norms between grades is likely to be much closer to random. Associations between these grade-specific gender norms and later outcomes are thus less likely to be due to unobserved confounders. We finally investigated the degree to which the gender expression of any individual differed from the norms within their proximal social environment. This difference measure offered us insight into whether diverging from the norm of the proximal environment comes at a cost, presumably due to perceived disapproval and possibly physical, verbal and/or social sanctioning. In all, these distinct measures provided us with a range of nuanced insights into how gender in this population impacts health and social mobility.

The flow chart in Fig. 1 could have helped us make the following decisions: Because the survey did not collect data about expectations of others, we had to develop a gender norms proxy. Because the many practices of interest were largely detectable, we clustered behaviour data at the PSU level (the school). Because the health concerns of interest (suicidal ideation, anti-social behaviours, etc.) were related to receiving sanctions from peers for not conforming with the gender norms of the cluster, we tested associations between non-conforming with the norms of the cluster and carrying out or experiencing the health-related negative outcomes of interest.

#### 2.1.5 Case study 5. Social network and community norms on adolescent childbirth in Honduras

Social network analysis provides an opportunity to more precisely – beyond that typically possible through cluster analysis – identify the reference group upholding norms that impact health. This can lead to important insights into targeted intervention strategies to shape gender norms and health.

##### 2.1.5.1 Motivation

Using data from a full population census and survey of the largely rural Copán department of Western Honduras, we analysed the social network determinants of adolescent childbirth at the individual level (Table 5). Data were collected as part of a randomised controlled trial of a method using social network targeting to implement a maternal and neonatal health intervention in a study area comprised of 176 villages (informal settlements of varying size) (Shakya et al., 2020). Adolescent childbearing in Central America, including in Honduras, is highly prevalent, with 25% of Honduran girls aged 15–19 either pregnant or a mother. Factors contributing to these high rates of adolescent childbearing are complex and include low social control of adolescent sexuality and injunctive norms around motherhood and fertility (Laplante et al., 2016). Girls who live in villages with a strong cultural emphasis on motherhood are at higher risk of adolescent childbearing while those who live in villages that hold strong norms against early pregnancy may be at lower risk (Denner et al., 2001). We hypothesised that norms expressed within social networks would be associated with risk of adolescent childbearing.

##### 2.1.5.2 Methodological roadblocks and solutions

Although the survey covered demographics, norms, and behaviours across the entire population, we limited our unit of analysis to girls between the ages of 15–20 for whom a childbirth event would have been within the past 3 years. We did this because our outcome, childbirth, preceded the survey itself. By keeping the timeframe relatively tight, we aimed to increase the likelihood that any normative associations we found could be reasonably tied to the recent childbirth event. Adolescent childbirth was assessed by asking girls whether or not they had given birth to a live child, and if so, the dates of birth of the last four children. Our normative and attitudinal measures included attitudes towards ideal age at first birth and injunctive norms on birth below the age of 18 which we included as individual-level measures and as village-level aggregate measures (villages were the primary sample units within the survey). The injunctive norms question asked all respondents residing within the sample villages: “If a girl younger than 18 has a baby, will people in the community think this is good, bad, or neither?” We categorised this as a binary outcome with good versus bad/neither as the statistical model showed no difference in the association between bad or neither with adolescent childbirth, but a strong difference between good and bad/neither. We created a village-level aggregate of adolescent childbirth among all female participants in the village, which we used as a proxy for descriptive norms.

Social network measures provide an overview of how norms, attitudes and behaviours are held across relationships between individuals. The data included social network connections between most individuals in the village, using a battery of questions designed to ascertain important relationships. All participants in each village were given a network survey; nominations could include anyone else residing within that same village. Participants were asked 14 separate name generator questions regarding their social connections to individuals within the community, including familial relationships, close personal relationships, economic support, and health advice. From these questions we were able to calculate individual network characteristics for each respondent based on the full network. Using this data we could understand reference groups at the wider community level, and at the more proximal interpersonal level using girls’ direct social ties. All of the questions asked of the adolescent girls in our study were also asked of everyone in the study population. This allowed us to match each adolescent with the social normative factors, including beliefs, attitudes, and adolescent birth outcome of each individual she nominated in the social network survey (alters), as well as with those who nominated her. Having 14 name generators meant that an individual could name any one alter up to 14 times so for any particular alter we also know in which questions they were named. Adding up the number of name generators through which a participant nominated a specific alter gave us a measure of tie strength, which can serve as a proxy for how close the participant feels to that particular alter. We also created a measure of direction reflecting whether the adolescent girl nominated the alter (out-ties), the alter nominated the girl (in-ties), or the nomination was reciprocal (both-ties). These measures allowed us to test the associations of attitudes and norms held by social contacts with the pregnancy outcomes of the individual girls they were socially connected to. For each girl, we were able to calculate which of her social contacts had been adolescent parents and included a descriptive norms proxy at the interpersonal level in addition to the village-level aggregate measure.

The flow chart in Fig. 1 could have helped us make the following decisions: Because the survey collected data about expectations of others, we clustered those data at the PSU level. Because the health concern of interest (early pregnancy) was related to carrying out a harmful action (early pregnancy) to meet approval, we tested the association between residing in a normative cluster and incurring an early pregnancy.

## 3 Discussion

Our intention is to offer methodological reflections and learnings that emerged from a cross-comparison of different approaches to investigate and quantify relationships between gender norms and health. We accept our explorations as preliminary, as there is still considerable path ahead in the investigation of how and to what extent gender norms affect health. However, we provide this synthesis to advance theory and stimulate further innovation in this field. Fig. 1 provides a decision-making flowchart for other researchers interested in measuring how gender norms affect people's lives in a context where gender norm data in largescale global health surveys is scarce. The flowchart was developed retrospectively, and as a way of illustrating its value, we offer ways in which the flowchart would have contributed to improved decision-making in the development of each case study.

Each case study underscores that collecting gender norms data would be highly valuable in global health surveys. When datasets include norms data, specifically asking participants what they believe others expect of them (as in the case of adolescents' norms related to body shape in Brazil and South Africa and in the case of injunctive norms about adolescent childbirth in Honduras), insights can generate unique findings without a reliance on proxies. This is important to inform the design of purposeful interventions with the potential for greater effectiveness in improving health outcomes under normative influence. Our case studies show the importance of collecting norms data from both adults and adolescents, as well as women, men and gender minorities to ensure the ability to infer expectations held by people of different genders and ages. Data need to be collected regularly to reveal trends of how gender norms are changing and to yield insights into their influence on people's health-related behaviours and health-related outcomes.

When gender norms data are not available, our methods for generating and using proxy measures can help others design effective analysis strategies. The case studies highlight a range of approaches to using individual-level data to generate aggregate proxy measures of norms at higher levels (e.g. parents, peer-group, social network, school, region). For example, using social network data in Honduras offered new avenues to explore how norms within smaller units like friendship circles and social networks affect health outcomes. In clustering individual data for use as a norms proxy, one can consider reported attitudes or reported or derived behaviours. To date, the most common solution to constructing norms proxies is to cluster attitudinal data (Legros and Cislaghi, 2019; Chung and Rimal, 2016). We suggest that the decision to cluster measures of attitudes and/or behaviours as proxies of gender norms should be taken while considering the contextual characteristics of the measure of interest. Clustering of attitudes might be better suited when the behaviour is difficult to detect (as in the case of premarital sex in Zambia) and when researchers can confidently suggest that people are likely to express their disapproval (so one person's attitudes give life to another person's injunctive norms). Clustering of behaviours might be better suited when the behaviour is performed visibly, as in the cases of women in Nigeria leaving the household to work, adolescent pregnancies in Honduras, and adolescents in US schools not conforming with gendered behaviours. Visible behaviours are detectable and can more easily result in sanctioning that will, in turn, shape people's injunctive norms (Cislaghi and Heise, 2018). Clustering of behaviours can also work to identify common behaviours for people who identify with a specific gender (as we did with Add Health data). Doing so can help identify people who act outside of that norm and enable examination of possible associations between acting outside of the norm and a variety of health-related and socio-economic outcomes. In the case of norms sanctioning the hidden, non-compliant act of premarital sex, we found that the discordance between professed attitudes and a calculated, rather than a reported measure of the behaviour shed new light on risk for HIV.

The Intraclass Correlation Coefficient (ICC), as used in case study 1 on IPV and labour in Nigeria, offers a promising analytical strategy to identify possible measures as valid proxies, beyond resorting to clustering attitudinal variables by default. We used data from the same dataset to validate the use of the technique. We tested the validity of the ICC using data on a practice well-known to be under normative influence – female genital mutilation/cutting (FGM/C) – and found that the ICC worked as hypothesised, providing results consistent with existing evidence (Wander and Shell-Duncan, 2020). Of course, the ICC does not necessarily flag the existence of a norm. High ICC levels might be due to other reasons, such as geographical features of the area where the data were collected (Legros and Cislaghi, 2019). The ICC, however, provides critical information to generate hypotheses about the possible existence of a norm, to be cross-referenced with the relevant mixed-methods literature.

Identifying possible sanctions following people's non-compliance with gender norms is another interesting avenue for further research into identifying health effects of norms. Health analysts can ground their investigation of the consequences of transgressing a gender norm by seeking to identify the upholders of norms, i.e., the reference group, and looking at possible penalties that they impose for failing to adhere to expected behaviours. We looked at partner violence as a possible sanction for women transgressing gender norms related to labour force participation, following the existing literature suggesting similar dynamics (Heise, 1998). We also found evidence that parents and peers can exert powerful sanctioning effects, even when their views are unstated but rather are perceived on the part of the transgressor of the norms.

We offer our methods and proxies in the hope that others will use, validate, and expand upon them in their future analyses. Doing so will advance understanding across settings and health outcomes with the potential of designing new research to explore pathways and hypotheses generated, and ultimately effective policies and programmes that both help achieve greater equality and improve people's health around the globe. Deeper understanding of pathways between norms and health outcomes may also further illuminate potentially powerful approaches to addressing gender norms that may simultaneously impact a multitude of health outcomes, as found for successful gender transformative programmes (Levy et al., 2020).

## Author statement

**Beniamino Cislaghi:** Conceptualization, Methodology, Investigation, Visualization, Writing – original draft; **Ann Weber:** Conceptualization, Formal analysis, Methodology, Writing – review & editing; **Holly Shakya:** Data curation, Formal analysis, Investigation, Writing – review & editing; **Safa Abdalla:** Data curation, Formal analysis, Investigation, Writing – review & editing; **Amiya Bhatia:** Visualization, Writing – original draft; **Benjamin Domingue:** Data curation, Formal analysis, Investigation, Writing – review & editing; **Iván Mejía-Guevara:** Data curation, Formal analysis, Investigation, Writing – review & editing; **Lindsay Stark:** Data curation, Formal analysis, Investigation, Writing – review & editing; **Ilana Seff:** Data curation, Formal analysis, Investigation, Writing – review & editing; **Linda M. Richter:** Data curation; **Ana Maria Baptista Menezes:** Data curation; **Cesar Victora:** Data curation; **Gary Darmstadt:** Conceptualization, Funding acquisition, Methodology, Project administration, Resources, Supervision, Visualization, Writing – original draft.

## Declaration of competing interest

BC, AMW, and AB were consultants on grant #OPP1140262 to 10.13039/100005492Stanford University from the 10.13039/100000865Bill and Melinda Gates Foundation. The authors have no other conflicts of interest to declare.

## Funding

10.13039/100000865Bill and Melinda Gates Foundation grant #OPP1140262 to 10.13039/100005492Stanford University.
