# Supplementary material for: Innovative methods to analyse the impact of gender norms on adolescent health using global health survey data
Source: Soc Sci Med. 2022 Jan;293:114652. doi: 10.1016/j.socscimed.2021.114652 (PMC8819155; doi:10.1016/j.socscimed.2021.114652)
Supplement: Multimedia component 1 [file mmc1.docx]

Supplemental Table 1. Measures and their corresponding questions used to create the GE variables:

Frequency of crying (0 = Never; 4 = Every day)

Frequency of playing an active sport (0 = Not at all; 3 = 5 or more times)

Got into a physical fight (0 = Never; 2 = More than once)

How do you think of yourself in terms of weight? (1 = Very underweight; 5 = Very overweight) How much do you feel that your friends care about you? (1 = Not at all; 5 = Very much)

Hours per week playing video/computer games (0–99 h)

What do you think your chances are of getting an STD? (1 = Very high; 5 = No chance)

How many hours do you spend working for pay (0–140 h)

Have you ever received an out-of-school suspension from school? (0 = No; 1 = Yes)

Frequency of poor appetite (0 = Never/Rarely; 3 = Most of the time)

Frequency of wearing a helmet while cycling (0 = Never; 4 = Always)

Hours per week listening to the radio (0–99 h)

Frequency of doing work around the house (0 = Not at all; 3 = 5 or more times)

Upset by difficult problems (1 = Strongly agree; 5 = Strongly disagree)

How much do you feel adults care about you? (1 = Not at all; 5 = Very much)

Frequency of moodiness (0 = Never; 4 = Every day)

You have a lot to be proud of (1 = Strongly agree; 5 = Strongly disagree)

Have you taken a pledge to remain a virgin until marriage? (0 = No; 1 = Yes)

Frequency of exercise (0 = Not at all; 3 = 5 or more times)

Rely on gut feelings to make decisions (1 = Strongly agree; 5 = Strongly disagree)

Trying to gain/lose/maintain weight? (1 = Lose; 4 = Nothing)
You never get sad (1 = Strongly agree; 5 = Strongly disagree)

How likely is it that you will go to college (1 = Low; 5 = High)

You felt you were just as good as other people (0 = Never/Rarely; 3 = Most of the time) Frequency wearing a seatbelt in the car (0 = Never; 4 = Always)
